# Prevalence, microbiological features, and clinical characteristics of *Elizabethkingia* isolates in a tertiary hospital, Jiangxi Province, China

**DOI:** 10.1016/j.imj.2025.100198

**Published:** 2025-08-19

**Authors:** Xiuhua Kang, Huaming Guo, Shanting Zhao, Wenzhen Zhang, Peng Liu, Yanfang Mei, Ling Zeng, Dandan Wei

**Affiliations:** a Jiangxi Provincial Key Laboratory of Prevention and Treatment of Infectious Diseases, Jiangxi Medical Center for Critical Public Health Events, The First Affiliated Hospital of Nanchang University, Nanchang 330052, Jiangxi Province, China; b Infection Control Division, The First Affiliated Hospital, Jiangxi Medical College, Nanchang University, Nanchang 330006, Jiangxi Province, China; c Medical College of Nanchang University, Nanchang 330006, Jiangxi Province, China; d Department of Pulmonary and Critical Care, The First Affiliated Hospital, Jiangxi Medical College, Nanchang University, Nanchang 330006, Jiangxi Province, China; e Department of Clinical Laboratory, The First Affiliated Hospital, Jiangxi Medical College, Nanchang University, Nanchang 330006, China

**Keywords:** *Elizabethkingia* isolates, Antimicrobial susceptibility, 16S rRNA gene sequencing, Risk factor

## Abstract

**Background:**

*Elizabethkingia* infections have become life-threatening hospital-acquired infections worldwide, marked by rising morbidity, multidrug resistance, and poor prognoses. However, information on the epidemiological and clinical characteristics of *Elizabethkingia* infections in mainland China is limited. This study aimed to analyze the molecular and clinical characteristics, and drug susceptibility of clinical *Elizabethkingia* isolates from a hospital in Jiangxi Province, China.

**Methods:**

A total of 103 *Elizabethkingia* isolates, identified by conventional methods, were collected from patients at a university-affiliated hospital in 2022 and 2023. Species identification was conducted using 16S rRNA gene sequencing. The feasibility of the Vitek MS was also evaluated. Antimicrobial susceptibility testing, resistance gene identification, and pulsed-field gel electrophoresis were performed.

**Results:**

The mean age of the patients was 61 years (excluding one 13-day-old infant) and 75.3 % were men. In total, 86.4 % of patients admitted to the intensive care unit were infected with *Elizabethkingia*. COVID-19, respiratory disease, and ICU admission were significantly different between the surviving and dying groups (*p* < 0.05). Sequencing of 103 isolates identified 92 strains of *Elizabethkingia anophelis*, eight strains of *Elizabethkingia meningoseptica,* two strains of *Elizabethkingia bruuniana*, and one strain of *Elizabethkingia ursingii.* The Vitek MS had a correct identification rate of 87 % for *Elizabethkingia anophelis*. More than 90 % of the *Elizabethkingia* isolates were susceptible to minocycline, but resistant to other drugs, including ceftazidime, aztreonam, and imipenem. Resistance genotype analysis showed that *blaBlaB* and *blaCME* were highly prevalent in the *Elizabethkingia* isolates. Molecular typing revealed 29 different pulsed-field gel electrophoresis types with clonal transmission between wards.

**Conclusions:**

Multidrug-resistant *Elizabethkingia* is being increasingly detected. Therefore, a larger database is required for *Elizabethkingia* strain identification. This database could be beneficial for the subsequent determination of optimal antimicrobial drugs for treating infections caused by various *Elizabethkingia* strains. Our pulsed-field gel electrophoresis model showed that most *Elizabethkingia* isolates exhibit sufficient genetic diversity and clonal transmission. Therefore, adequate attention should be directed towards this pathogen.

## Highlights

• Elderly male ICU patients predominant in *Elizabethkingia* infection cases.
• COVID-19 and respiratory diseases correlate with higher mortality risk.
• Minocycline shows > 90 % susceptibility against multidrug-resistant *Elizabethkingia* isolates.
• Clonal transmission observed among genetically diverse *Elizabethkingia* strains in hospital wards.

## Graphical abstract

**Figure:**
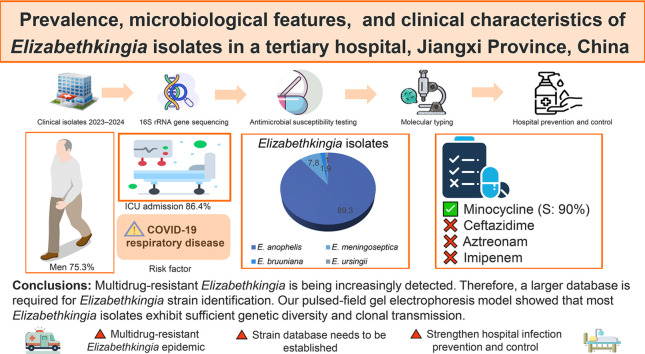

## 1 Introduction

The genus *Elizabethkingia* consists of aerobic, oxidase-positive, glucose-unfermenting, nonautotrophic, Gram-negative bacilli that are common in soil, freshwater, saltwater, and hospital environments, but rare in humans.^1^^,^^2^ Although a rare pathogen, *Elizabethkingia meningoseptica*, which causes neonatal meningitis outbreaks, causes life-threatening infections and is associated with human infections since it was first reported by Elizabeth O. King in 1959 in a neonatal case of meningitis.^3^^,^^4^ Advances in molecular techniques have revealed that several isolates, previously classified as *E. meningoseptica,* belong to different species with new classifications and nomenclature. To date, at least seven species, namely, *E. meningoseptica, Elizabethkingia anophelis*,^5^ *Elizabethkingia miricola*,^6^ *Elizabethkingia argenteiflava*,^7^ *Elizabethkingia occulta, Elizabethkingia ursingii*, and *Elizabethkingia bruuniana*,^8^ have been classified into the genus *Elizabethkingia. E. anophelis* was isolated from the midgut of a mosquito (*Anophelis gambiae*) in 2011.^7^ The first documented human infection of *E. anophelis* occurred in 2013, involving a case of meningitis in a newborn in the Central African Republic.^9^ In 2018, three new species were identified: *E. occulta, E. ursingii,* and *E. bruuniana*.^10^

Environmental studies have shown that *Elizabethkingia* can survive in water supply systems and often colonizes sinks, basins, and faucets, creating a potential reservoir of infection within hospitals.^11^ *Elizabethkingia* can be introduced into patients through medical equipment contaminated with fluids (e.g., respirators, intubation tubes, fog tents, humidifiers, neonatal incubators, and freezers), and can also be transmitted through wet and dry materials and surfaces, including the hands of hospital staff. Hospital transmission of *Elizabethkingia* has also been reported in immunocompromised adults in intensive care units (ICUs). Nosocomial outbreaks of *Elizabethkingia* occur worldwide, especially through infections in ICU patients requiring ventilator support.^2^ Outbreaks have been mainly related to healthcare and, often, water sources.^12^^,^^13^ Evidence suggests that most infections in humans are caused by *E. anophelis*.^14^

The increasing number of *Elizabethkingia* infections worldwide in recent years, with high morbidity and mortality rates, highlights the importance of early detection and treatment.^3^ Due to their Ambler Class A serine extended-spectrum β-lactamase gene, *blaCME*, and the Ambler Class B meta llo-β-lactamase genes, *blaBlaB* and *blaGOB, Elizabethkingia* species are intrinsically resistant to a wide variety of β-lactams, contributing to their natural resistance to several commonly used carbapenem antibiotics. *Elizabethkingia* species are resistant to quinolones, owing to DNA mutations in their rotamase and/or topoisomerase IV genes.^15^^,^^16^ *Elizabethkingia* has the unique ability to acquire multi-drug resistance and survive disinfectants. Therefore, its spread between patients via human/inanimate host material in hospital environments is a concern. Thus, it is critical to identify the source of infection and establish the kinetics of its spread within hospital environments.^3^ *Elizabethkingia*-related infections are complicated by biofilm formation, intracellular invasion, and multidrug resistance of strains, necessitating the careful selection of appropriate antimicrobial agents.

Three species, *E. meningoseptica, E. miricola,* and *E. anophelis*, cannot be distinguished by their phenotypic characteristics, and are often misidentified by biochemical or other commercial systems because of the limited *Elizabethkingia* database. Previous studies have misidentified *E. anophelis, E. bruniana, E. ursingii*, and *E. occulta* as *E. meningoseptica*, suggesting an underestimation of the likelihood of infection with these species.^10^^,^^16^, ^17^, ^18^, ^19^ Most studies investigating *Elizabethkingia* have used unreliable microbial identification methods.^17^ Therefore, these studies present the clinical or molecular characteristics of all *Elizabethkingia* species rather than focusing on individual species. Despite their clinical significance, gaps remain in our understanding of the demographics, pathogenicity, and effective treatment options of *Elizabethkingia* infections.

In this study, we analyzed the epidemiology, clinical characteristics, and antibiotic susceptibility of *Elizabethkingia* isolates collected from the First Affiliated Hospital of Nanchang University in 2022 and 2023 using 16S rRNA sequencing. We evaluated the susceptibility of *Elizabethkingia* isolates to 16 antibiotics and compared the results of 16S rRNA sequencing with those of the VITEK MS assay to identify the strains to evaluate the feasibility of this mass spectrometry.

## 2 Materials and methods

### 2.1 Clinical specimens and identification of Elizabethkingia isolates

Clinical isolates for bacterial culture were collected from the First Affiliated Hospital of Nanchang University, a tertiary comprehensive hospital in China with 6100 beds, between January 2022 and December 2023. A total of 103 clinical isolates were collected from 81 hospitalized patients, either from different sources or from multiple isolates obtained during the hospitalization of the same patient. The species were initially identified using matrix-assisted laser desorption ionization-time of flight mass spectrometry (MALDI-TOF MS) (VITEK MS; bioMérieux, Marcy l’Étoile, France). The isolates identified as *Elizabethkingia spp*. were frozen until use.

### 2.2 Species identification using 16S rRNA gene sequencing

A total of 103 *Elizabethkingia* clinical isolates from various specimens of hospitalized patients. Initial species identification was conducted using Vitek MS and genetically confirmed by 16S rRNA sequencing using the following previously used universal primers: 27F, 5′-AGAGTTTGATCMTGGCTCAG-3′ and 1492R, 5′-TACGGYTACCTTGTTACGACTT-3′.^16^ The assembled 16S rRNA sequences were submitted to the National Center for Biotechnology Information website for comparison with the GenBank sequence database using the Basic Local Alignment Search Tool (https://blast.ncbi.nlm.nih.gov/Blast.cgi). The similarity of the 16S rRNA sequences of isolates to the type strains in the GenBank sequence databases was examined using the following reference sequences: *E. anophelis* strain R26, GenBank accession number NR_116,021.1; *E. meningoseptica* type strain 13,253, NR_042267.1; *E. bruuniana* strain SBRL-21–126, NZ_JAMBNJ010000000; *E. ursingii* strain G4122, NZ_LNOK01000023.^20^ All clinical isolates were identified by Vitek MS and 16S rRNA gene sequencing, and with 16S rRNA as a reference, we compared the accuracy of Vitek MS in identifying *Elizabethkingia* isolates.^21^

### 2.3 Antimicrobial susceptibility testing

*In vitro* drug susceptibility testing was conducted using the Vitek2-Compact fully automated microbial analysis system (bioMérieux, Marcy L'Étoile or Craponne, France). Interpretations of resistance (R), intermediate resistance (I), and sensitivity (S) were performed in accordance with the criteria established by the Clinical Laboratory Standards Institute (M100-S27, https://iacld.com/UpFiles/Documents/672a1c7c-d4ad-404e-b10e-97c19e21cdce.pdf). PCR amplification was performed to detect the presence of seven resistance genes (*blaBlaB, blaGOB, blaCME, GryA, GyrB, ParC*, and *ParE*), as previously described.^18^ The amplification primers, systems, and conditions were obtained from the literature.

### 2.4 Molecular typing

Pulsed-field gel electrophoresis (PFGE) was used to appraise the homology of all strains.^22^ Genomic DNA of *Elizabethkingia* was fabricated via digestion with the restriction enzyme XhoI (Takara Bio Inc., Shiga, Japan)^22^^,^^23^ for 4 h at 37 °C. The molecular size marker of strain Braenderup H9812 was processed with XbaI (Takara Bio Inc., Shiga, Japan) .^11^ Furthermore, the DNA fragments were segregated using the CHEF Mapper XA System (Bio-Rad Hercules, CA, USA) at 6 V/cm for 18 h. PFGE band profiles were analyzed with BioNumerics 8.0 (Applied Maths, Sint-Martens-Latem, Belgium). Similarity matrices were computed using Dice's coefficients with 1.5 % optimization and 1.5 % band matching tolerance. Dendrograms were constructed using the unweighted pair group method with arithmetic averages.^24^ Isolates were categorized into PFGE subtypes (≥ 95 % similarity), PFGE types (85 % to less than 95 % similarity), or different types (< 85 % similarity).^21^

### 2.5 Statistical analysis

Data were analyzed using IBM SPSS Statistics for Windows, version 26 (IBM Corp., Armonk N.Y., USA). Categorical data are expressed as frequencies and percentages. Chi-squared or Fisher's exact tests were used to compare categorical variables (sex, underlying diseases, operation, indwelling device, ICU admission, principal disease, fungal infection, and COVID-19). Continuously quantitative data (age, hospitalization duration, temperature, white blood cell count, hemoglobin, neutrophil percentage, platelet count, lymphocyte count, lymphocyte percentage, and levels of procalcitonin, C-reactive protein, and serum creatinine) are expressed as the mean ± standard deviation and compared using Student's *t-*test. A *p*-value of < 0.05 was considered significant difference.

## 3 Results

### 3.1 Identification and prevalence of Elizabethkingia isolates

A total of 103 *Elizabethkingia* isolates, identified using conventional methods, were collected at a university-affiliated hospital in 2022 and 2023. Of the 103 isolates, using 16S rRNA gene sequencing, 92 (89.3 %) were identified as *E. anophelis* (99.4 %–100.0 % nucleotide identity to *E. anophelis* type strain R16), eight (7.8 %) as *E. meningoseptica* (99.5 %–99.9 % nucleotide identity to *E. meningoseptica* type strain ATCC 13,253), two (1.9 %) as *E. bruuniana,* and one (1.0 %) as *E. ursingii*. However, ambiguity was noted in the identification of *E. bruuniana* and *E. ursingii*.

MALDI-TOF MS with an amended database was used and its feasibility for the identification of *Elizabethkingia* isolates was evaluated. Using VITEK MS, 80.6 % of *Elizabethkingia* isolates (83 of 103) were correctly identified. VITEK MS identified 80 strains (77.7 %) of *E. anophelis* and correctly identified three strains (2.9 %) of *E. meningoseptica*, demonstrating better accuracy compared with other methods. Of these, seven (6.8 %) strains of *E. anophelis* were misidentified as *E. miricola*, five (4.8 %) strains of *E. anophelis* were misidentified as *E. meningoseptica*, five (4.8 %) strains of *E. meningoseptica* were misidentified as *E. anophelis*, and one (1.0 %) strain of *E. ursingii* was misidentified as *E. anophelis*. Furthermore, there was one instance each of *E. bruuniana* being misidentified as *E. miricola* (1.0 %) and *E. bruuniana* as *E. anophelis* (1.0 %). These results imply that Vitek MS may be unreliable in identifying *E. meningoseptica* and *E. miricola*. Additionally, 16 sputum samples showed concomitant isolates of other bacterial species, such as *Acinetobacter baumannii, Acinetobacter SPP, Klebsiella pneumoniae,* and *Stenotrophomonas maltophilia*.

During the 2-year study period involving 317,301 hospitalized patients, 81 of them were identified as having been infected with *Elizabethkingia*, which led to a prevalence of 2.55 per 10,000. In 2022, 30 patients were screened out from 137,067 hospitalized patients, and in 2023, 51 patients were screened out from 183,725 hospitalized patients. The prevalence went up from 2.19 per 10,000 to 2.78 per 10,000. A review of the number of Gram-negative bacilli over the two-year period 2022–2023 revealed a total of 9363 cases, with *Elizabethkingia* strains accounting for 1.1 % of the total Gram-negative bacilli.

### 3.2 Clinical characteristics of Elizabethkingia infections

**Fig. 1:**
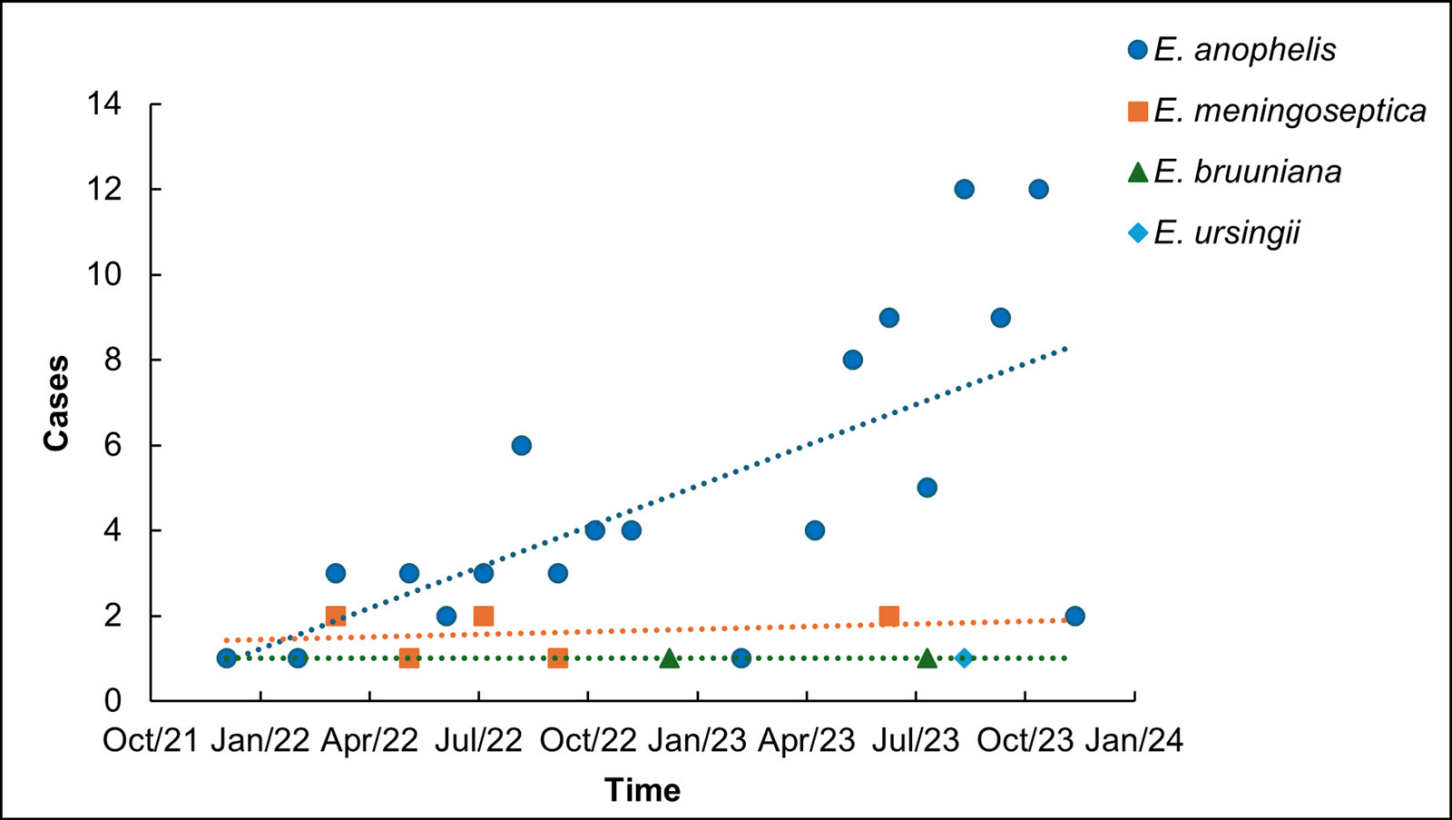
Isolate count of *Elizabethkingia* from January 2022 to December 2023.

**Fig. 2:**
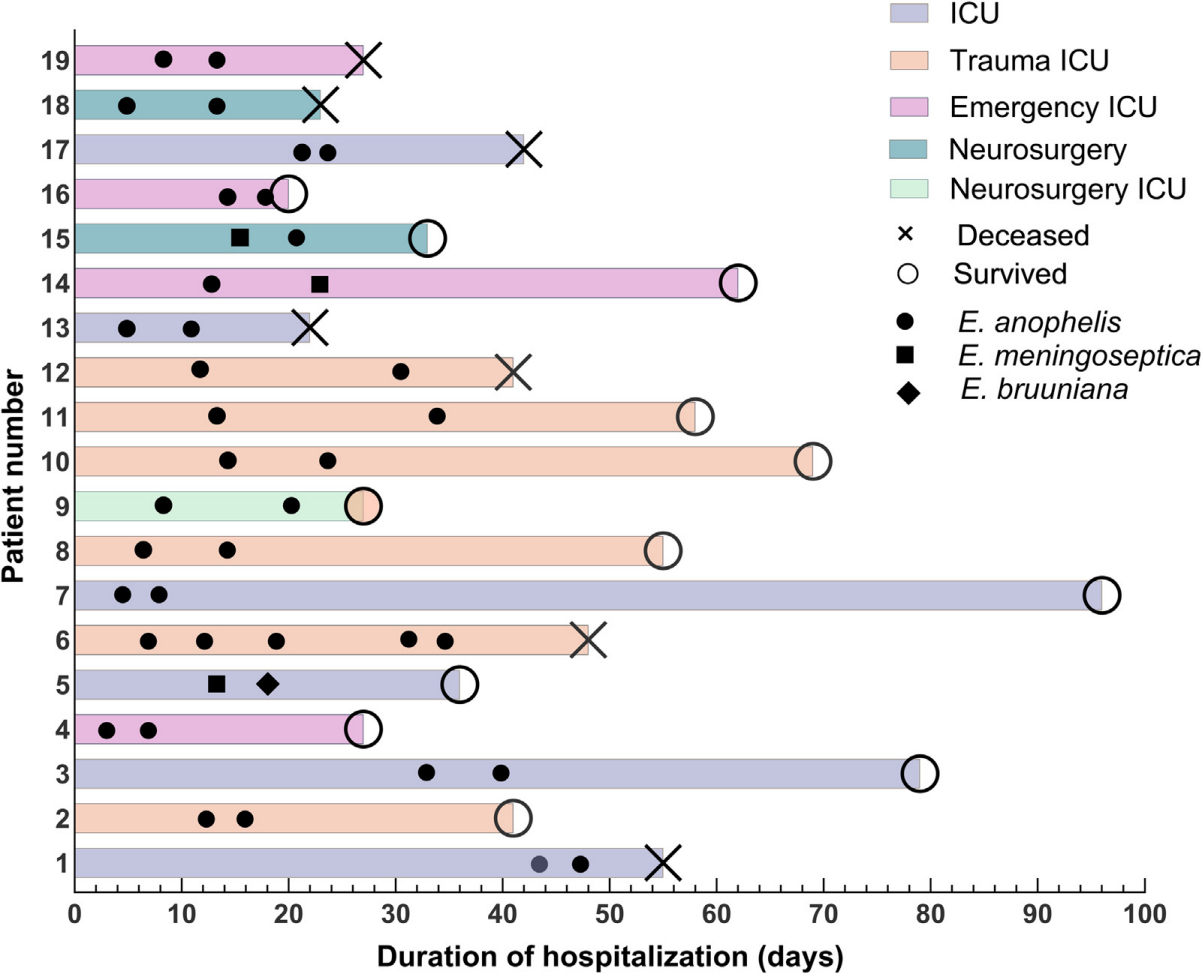
Baseline characteristics of the 19 patients with recurrent isolates. The X-axis depicts time since hospital admission, while the Y-axis represents baseline characteristics of the 19 patients. Ward types are denoted by solid squares of distinct colors. Among these patients, 7 were deceased and 12 survived.

*Abbreviation*: ICU, intensive care unit.

During 2022–2023, 103 *Elizabethkingia spp*. isolates were collected (36 in 2022 and 67 in 2023), demonstrating a significant upward trend in overall isolation rates. *E. anophelis* exhibited the most pronounced increase in detection frequency (Fig. 1). Respiratory tract specimens constituted the primary isolation source (90.3 %), followed by bloodstream infections (3.9 %). Other isolation sites included cerebrospinal fluid (1.9 %), urine (1.9 %), pleural fluid (1.0 %), and catheter tips (1.0 %). Among the 103 *Elizabethkingia* isolates included in the study, 8 were identified as *E. meningoseptica*. Patients infected with this species had no history of COVID-19 and demonstrated significantly lower frequencies of underlying comorbidities—including shock, malignancies, and respiratory/digestive disorders—compared to those with *E. anophelis* infections. These findings suggest that patients infected with *E. anophelis* may present a heightened comorbidity burden (Table S1). A total of 103 *Elizabethkingia spp*. isolates were recovered from 81 patients through multi-site sampling or serial collections. Eighteen patients exhibited recurrent isolations (twice), while one patient had five isolations (Fig. S1). Most recurrent isolations occurred within ≤ 5-day intervals. Eighteen patients had ICU hospitalization history, with several cases showing different species isolated from the same patient (e.g., *E. anophelis* and *E. meningoseptica*; Fig. 2), a phenomenon requiring further investigation. Notably, sputum cultures from two patients in the trauma ICU concurrently yielded *E. anophelis* during the same period. The recurrent isolation patterns—both intra-patient and inter-patient—indicate ICUs constitute high-risk zones. This necessitates enhanced disinfection and surveillance of medical devices, environmental surfaces, and healthcare workers' hands, coupled with strict adherence to aseptic protocols to prevent cross-transmission.

Of these patients, 75.3 % were men and 24.7 % were women; the average age of the patients was 61 ± 19 years (excluding one 13-day-old patient) (Table 1). Prolonged hospital stays (≥ 2 weeks) were observed in 75 patients. Comorbidities were identified in most hospitalized patients, with hypertension being the most prevalent underlying disease (31/81; 38.3 %), followed by diabetes mellitus (15/81; 18.5 %), and chronic obstructive pulmonary disease (19/81; 23.5 %). A large portion of the patients had nervous system disease (55.6 %), while 54.3 % had cardiovascular disease, and 35.8 % had experienced trauma. Furthermore, 70 (86.4 %) patients were treated in the ICU, 61 (75.3 %) underwent surgery, and 63 (77.8 %) received mechanical ventilation. Central venous catheters were placed in 58 patients (71.6 %).

**Table 1:** Factors associated with mortality in patients with *Elizabethkingia* infections.

	Total (*n* = 81)	Survivors (*n* = 54)	Deaths (*n* = 27)	*z/χ^2^*	*p*
Age, years, median (IQR)	60.0 (53.5, 74.5)	57.5 (50.0, 69.5)	72.0 (58.0, 81.0)	−3.002	0.003
Age ≥ 65, years, *n* (%)	36 (44.4)	19 (35.2)	17 (63.0)	5.625	0.018
Male, *n* (%)	61 (75.3)	39 (72.2)	22 (81.5)	0.830	0.362
Hospitalization duration, days, median (IQR)	36.0 (22.0, 54.0)	37.0 (22.8,62.5)	25.0 (21.0, 45.0)	−1.579	0.114
Operation, *n* (%)	61 (75.3)	42 (77.8)	19 (70.4)	0.531	0.466
Indwelling device, *n* (%)					
Mechanical ventilation	63 (77.8)	40 (74.1)	23 (85.2)	0.723	0.395
Central venous catheter	58 (71.6)	35 (64.8)	23 (85.2)	2.740	0.098
Nasogastric tube	58 (71.6)	35 (64.8)	23 (85.2)	2.740	0.098
Foley's catheter	60 (74.1)	37 (68.5)	23 (85.2)	1.808	0.179
Surgical puncture or drain	33 (40.7)	21 (38.9)	12 (44.4)	0.230	0.631
ICU admission, *n* (%)	70 (86.4)	43 (79.6)	27 (100.0)	−	0.013
COVID-19, *n* (%)	10 (12.3)	2 (3.7)	8 (29.6)	11.180	0.001
Fungal infection, *n* (%)	32 (39.5)	18 (33.3)	14 (51.9)	2.583	0.108
Underlying diseases, *n* (%)					
Diabetes mellitus	15 (18.5)	10 (18.5)	5 (18.5)	0.000	1.000
Hypertension	31 (38.3)	20 (37.0)	11 (40.7)	0.105	0.746
Chronic obstructive pulmonary disease	19 (23.5)	8 (14.8)	11 (40.7)	6.739	0.009
Principle disease, *n* (%)					
Nervous system	45 (55.6)	28 (51.9)	17 (63.0)	0.900	0.343
Malignancy	5 (6.2)	4 (7.4)	1 (3.7)	0.027	0.870
Trauma	29 (35.8)	23 (42.6)	6 (22.2)	3.250	0.071
Cardiovascular	44 (54.3)	28 (51.9)	16 (59.3)	0.398	0.528
Digestive	13 (16.0)	11 (20.4)	2 (7.4)	1.386	0.239
Respiratory	33 (40.7)	17 (31.5)	16 (59.3)	5.753	0.016
Temperature, °C, median (IQR)	37.7(36.9, 38.3)	36.8(37.7, 38.1)	37.7(36.9, 38.5)	−0.582	0.561
Laboratory data, median (IQR)					
White blood cell count, × 10^9^/L	10.2 (7.5, 14.6)	9.5 (7.4, 14.0)	12.7 (8.7, 17.3)	−1.733	0.083
Hemoglobin, g/L	87.0 (74.0, 97.0)	88.5 (75.0, 97.8)	82.0 (72.0, 97.0)	−1.218	0.223
Platelet count, × 10^9^/L	221.0 (65.0, 314.5)	249.5 (140.3, 328.5)	137.0 (56.0, 276.0)	−2.034	0.042
Neutrophil percentage, %	83.0 (75.9, 88.0)	82.0 (74.2, 85.8)	84.3 (78.4, 91.9)	−2.129	0.033
Lymphocyte count, × 10^9^/L	0.9 (0.6, 1.4)	1.0 (0.6, 1.3)	0.8 (0.2, 1.5)	−0.676	0.499
Lymphocyte percentage, %	9.5 (4.9, 13.1)	10.0 (5.4, 13.2)	5.4 (3.4, 13.1)	−1.864	0.062
C-reactive protein, mg/L	55.8 (21.7, 94.6)	44.2 (19.7, 81.0)	94.6 (38.4, 145.5)	−3.086	0.002
Serum creatinine, mg/dL	79.1(55.5, 119.0)	70.0 (52.5, 109.0)	114.7 (75.9, 182.4)	−2.675	0.007
Procalcitonin, ng/mL	0.6 (0.2, 2.5)	0.4 (0.2, 1.2)	1.5 (0.5, 5.4)	−3.014	0.003

*Abbreviation*: IQR, interquartile range.

A total of 27 deaths occurred, corresponding to a mortality rate of 33.3 %. Compared to the survivors, the 27 patients who died were significantly older 72.0 (interquartile range [IQR] 58.0–81.0) vs. 57.5 [IQR 50.0–69.5] years; *p* = 0.003) and had a higher proportion of elderly patients (≥ 65 years: 63.0 % vs. 35.2 %; *p* = 0.018). Furthermore, the distribution of primary diseases showed a significant difference, with a higher percentage of respiratory diseases in patients who died compared to survivors (59.3 % vs. 31.5 %; *p* = 0.016). COVID-19 was the most significant risk factor associated with mortality (8 % vs. 2 %; *p* = 0.001). C-reactive protein, serum creatinine, neutrophil percentage, platelet count, and procalcitonin levels were significantly different between the survival and death groups (*p* < 0.05), whereas white blood cell count, hemoglobin level, lymphocyte count, and lymphocyte percentage showed no significant differences between the groups. Multivariate analysis identified no independent predictors of in-hospital mortality; however, concomitant COVID-19 infection was demonstrated as a significant predictor of death (Table S2).

### 3.3 Antimicrobial susceptibilities and genotype of Elizabethkingia isolates

The drug susceptibilities of the 103 *Elizabethkingia* isolates were determined using the Vitek2-Compact fully automated microbial analysis system (Table 2). All isolates were resistant to aztreonam. The majority of the isolates were susceptible to minocycline (97 %), followed by doxycycline (89 %) and trimethoprim-sulfamethoxazole (81 %). More than 95 % of the tested isolates were resistant to ceftazidime, imipenem, meropenem, amikacin and tobramycin. Additionally, one *E. anophelis* isolate was resistant to all antibiotics tested; however, most *Elizabethkingia* isolates were only sensitive to two or three antibiotics tested.

**Table 2:** Antimicrobial susceptibilities of 103 *Elizabethkingia* isolates determined by the Vitek2-Compact fully automated microbial analysis system.

No. of isolates with result/Total No. of isolates tested (%)															
	*E. Anophelis*			*E. Meningoseptic*			*E. Bruuniana*			*E. Ursingii*			Total isolates		
Antimicrobial agents	S	I	R	S	I	R	S	I	R	S	I	R	S	I	R
Piperacillin-tazobactam	26/90 (28.9)	3/90 (3.3)	61/90 (67.8)	6/8 (75.0)	0 (0.0)	2/8 (25.0)	0 (0.0)	1/2 (50.0)	1/2 (50.0)	0 (0.0)	0 (0.0)	1/1 (100.0)	32/101 (31.7)	4/101 (4.0)	65/101 (64.3)
Ticarcillin-clavulanic acid	2/55 (3.6)	6/55 (10.9)	47/55 (85.5)	0 (0.0)	0 (0.0)	2/2 (100.0)	0 (0.0)	0 (0.0)	1/1 (100.0)	0 (0.0)	0 (0.0)	1/1 (100.0)	2/59 (3.4)	6/59 (10.2)	51/59 (86.4)
Ceftazidime	0 (0.0)	1/68 (1.5)	67/68 (98.5)	0 (0.0)	0 (0.0)	2/2 (100.0)	0 (0.0)	0 (0.0)	2/2 (100.0)	0 (0.0)	0 (0.0)	1/1 (100.0)	0 (0.0)	1/73 (1.4)	72/73 (98.6)
Cefepime	0 (0.0)	5/91 (5.5)	86/91 (94.5)	0 (0.0)	0 (0.0)	8/8 (100.0)	0 (0.0)	0 (0.0)	2/2 (100.0)	0 (0.0)	0 (0.0)	1/1 (100.0)	0 (0.0)	5/102 (4.9)	97/102 (95.1)
Cefoperazone-sulbactam	2/16 (12.5)	2/16 (12.5)	12/16 (75.0)	0 (0.0)	0 (0.0)	1/1 (100.0)	0 (0.0)	0 (0.0)	1/1 (100.0)	0 (0.0)	0 (0.0)	0 (0.0)	2/18 (11.1)	2/18 (11.1)	14/18 (77.8)
Aztreonam	0 (0.0)	0 (0.0)	91/91 (100.0)	0 (0.0)	0 (0.0)	8/8 (100.0)	0 (0.0)	0 (0.0)	2/2 (100.0)	0 (0.0)	0 (0.0)	1/1 (100.0)	0 (0.0)	0 (0.0)	102/102 (100.0)
Imipenem	2/92 (2.2)	0 (0.0)	90/92 (97.8)	0 (0.0)	0 (0.0)	8/8 (100.0)	0 (0.0)	0 (0.0)	2/2 (100.0)	0 (0.0)	0 (0.0)	1/1 (100.0)	2/103 (1.9)	0 (0.0)	101/103 (98.1)
Meropenem	0 (0.0)	2/62 (3.2)	60/62 (96.8)	0 (0.0)	0 (0.0)	2/2 (100.0)	0 (0.0)	0 (0.0)	1/1 (100.0)	0 (0.0)	0 (0.0)	1/1 (100.0)	0 (0.0)	2/66 (3.0)	64/66 (97.0)
Amikacin	3/92 (3.3)	1/92 (1.1)	88/92 (95.6)	0 (0.0)	0 (0.0)	8/8 (100.0)	1/2 (50.0)	0 (0.0)	1/2 (50.0)	0 (0.0)	0 (0.0)	1/1 (100.0)	4/103 (3.9)	1/103 (1.0)	98/103 (95.1)
Ciprofloxacin	21/92 (22.8)	3/92 (3.3)	68/92 (73.9)	1/8 (12.5)	0 (0.0)	7/8 (87.5)	2/2 (100.0)	0 (0.0)	0 (0.0)	0 (0.0)	0 (0.0)	1/1 (100.0)	24/103 (23.3)	3/103 (2.9)	76/103 (73.8)
Levofloxacin	30/92 (32.6)	0 (0.0)	62/92 (67.4)	1/8 (12.5)	0 (0.0)	7/8 (87.5)	1/2 (50.0)	0 (0.0)	1/2 (50.0)	0 (0.0)	0 (0.0)	1/1 (100.0)	32/103 (31.1)	0 (0.0)	71/103 (68.9)
Trimethoprim-sulfamethoxazole	71/89 (79.8)	0 (0.0)	18/89 (20.2)	8/8 (100.0)	0 (0.0)	0 (0.0)	1/2 (50.0)	0 (0.0)	1/2 (50.0)	1/1 (100.0)	0 (0.0)	0 (0.0)	81/100 (81.0)	0 (0.0)	19/100 (19.0)
Doxycycline	54/61 (88.5)	1/61 (1.6)	6/61 (9.8)	2/2 (100.0)	0 (0.0)	0 (0.0)	1/1 (100.0)	0 (0.0)	0 (0.0)	1/1 (100.0)	0 (0.0)	0 (0.0)	58/65 (89.3)	1/65 (1.5)	6/65 (9.2)
Minocycline	59/61 (96.7)	0 (0.0)	2/61 (3.3)	2/2 (100.0)	0 (0.0)	0 (0.0)	1/1 (100.0)	0 (0.0)	0 (0.0)	1/1 (100.0)	0 (0.0)	0 (0.0)	63/65 (96.9)	0 (0.0)	2/65 (3.1)
Gentamicin	1/31 (3.2)	4/31 (12.9)	26/31 (83.9)	0 (0.0)	0 (0.0)	6/6 (100.0)	0 (0.0)	0 (0.0)	1/1 (100.0)	0 (0.0)	0 (0.0)	0 (0.0)	1/38 (2.6)	4/38 (10.5)	33/38 (86.8)
Tobramycin	3/92 (3.3)	0 (0.0)	89/92 (96.7)	0 (0.0)	0 (0.0)	8/8 (100.0)	1/2 (50.0)	0 (0.0)	1/2 (50.0)	0 (0.0)	0 (0.0)	1/1 (100.0)	4/103 (3.9)	0 (0.0)	99/103 (96.1)

**Fig. 3:**
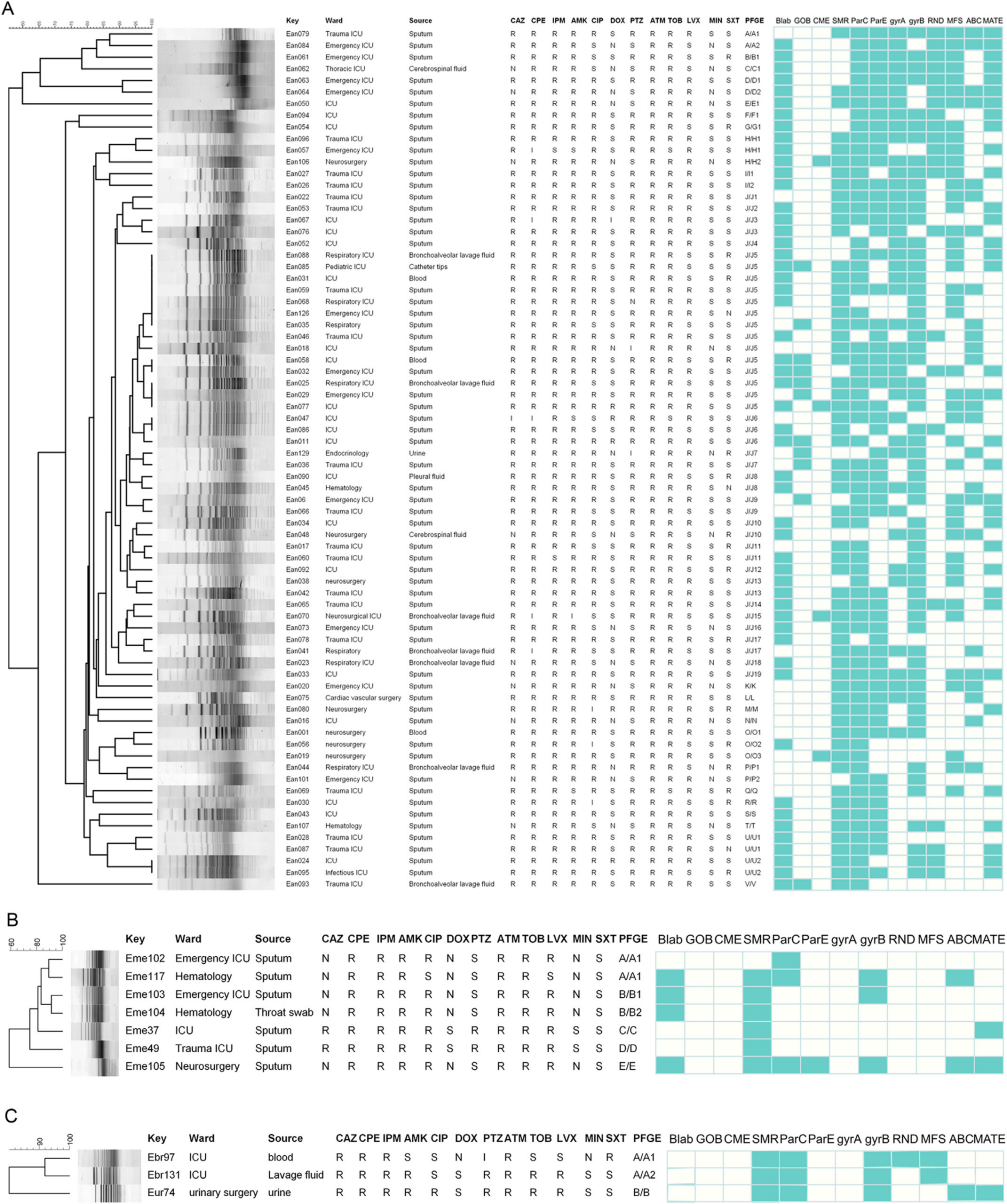
Dendrogram of PFGE patterns of 84 *Elizabethkingia* isolates using the BioNumerics software. (A) Seventy-four *E. anophelis* isolates; (B) Seven *E. meningoseptica* isolates; (C) Two *E. bruuniana* and one *E. ursingii* isolates.

*Abbreviations*: M, male; F, female; CAZ, ceftazidime; CPE, cefepime; IPM, imipenem; AMK, amikacin; CIP, ciprofloxacin; DOX, doxycycline; PTZ, piperacillin tazobactam; ATM, aztreonam; TOB, tobramycin; LVX, levofloxacin; MIN, minocycline; SXT, sulfamethoxazole; S, susceptible; I, intermediate; R, resistant.

A total of 69 *Elizabethkingia* strains carried β-lactamase genes. Of these, 68 *Elizabethkingia* isolates carried *blaBlaB* and seven carried *blaCME*; none carried *blaGOB*. Six *Elizabethkingia* isolates harbored both *blaBlaB* and *blaCME* genes (Fig. 3). Accordingly, strains carrying these resistance genes were resistant to ceftazidime, cefepime, meropenem, and imipenem, with resistance rates > 90 %; This effect was particularly pronounced in *E. anophelis* and *E. meningoseptica*. Of the 103 strains of *Elizabethkingia*, the strains that were positive for *GryA, GyrB, ParC,* and *ParE* showed resistance to fluoroquinolones, and the sensitivity rate of ciprofloxacin was approximately 30 %. Furthermore, the strains carrying the *resistance-nodulation-cell division* (*RND*) gene were more susceptible to ciprofloxacin.

### 3.4 Molecular typing of Elizabethkingia isolates

Nineteen *Elizabethkingia* isolates were resistant to XhoI digestion. The remaining 84 isolates clustered into 29 different PFGE types (Fig. 3). In particular, 74 *E. anophelis* isolates were divided into 22 clusters designated A–V, seven *E. meningoseptica* isolates were divided into five clusters designated A–E, and two *E. bruuniana* and one *E. ursingii* isolate were divided into two clusters designated A and B. PFGE typing was most common for the J-type with 42 strains, 14 of which belonged to the same subtype. All of these 14 strains were identified in patients admitted to the ICU of the same department within an eight-month span. Clonal strains of the same subtype, primarily collected from sputum, were detected, indicating clonal transmission in the ICU. Most patients with the same subtype experienced cerebrovascular accidents, and received mechanical ventilation and indwelling tubes during hospitalization. Similar antimicrobial susceptibility patterns were observed for different subtypes of the same clustered strains.

## 4 Discussion

*Elizabethkingia* isolates cause serious nosocomial infections and outbreaks worldwide, but have received relatively little attention. In this study, we used 16S rRNA gene sequencing as a reference method for the species identification of *Elizabethkingia spp*. collected over two years and analyzed the characteristics of *Elizabethkingia spp*. obtained from clinical samples. We found that *E. anophelis*, but not *E. meningoseptica*, accounted for the majority of human infections with the genus *Elizabethkingia,* and that the isolation rate of *Elizabethkingia* almost doubled from 36 strains collected in 2022 to 67 strains in 2023, with increasing detection of *E. anophelis*. Evidence suggests that *E. anophelis,* rather than *E. meningoseptica,* dominates *Elizabethkingia* in clinical settings.^18^ Therefore, nosocomial *Elizabethkingia* infections should be further studied.

Advances in microbial identification techniques have enabled the identification of several emerging unusual bacteria that cause disease, primarily in immunocompromised patients. Traditional identification systems are poor at identifying rare species and can easily lead to misidentification, misdiagnosis, treatment failure, and underestimation of the incidence of infection.^25^ MALDI-TOF, Vitek mass spectrometry, and molecular identification techniques (16S rRNA, *rpoB* gene sequencing, and whole genome sequencing) have become useful tools for the accurate identification of microorganisms.^15^^,^^21^ These tools have an excellent discrimination ability, especially for rare opportunistic bacteria. In this study, we found ambiguity in the identification of *Ebr97, Ebr131*, and *Eur74* by 16S rRNA gene sequencing, which may be attributed to the presence of multiple copies of the different sequences, as well as the fact that these are highly variable regions of 16S rRNA.^3^ PFGE mapping showed better resolution of clonal relationships, indicating that *Ebr131* is more closely related to *Ebr97*, with a similarity of 85.7 %. The *rpoB* gene is a single-copy gene with a higher phylogenetic evolutionary resolution than the 16S rRNA genes, thus allowing for accurate differentiation of *Elizabethkingia* at the species level.^10^^,^^15^

In contrast to previous studies, our investigation revealed that *Elizabethkingia* was isolated on multiple occasions during the course of hospitalization in 19 out of 81 patients. Moreover, the majority of these isolations were obtained on multiple occasions within a single week, suggesting that the strain is recalcitrant to clearance. Consequently, it is imperative to monitor for resistance and virulence traits associated with this strain. Additionally, we discovered that multiple isolates of *Elizabethkingia* from the same patient were identified as different species, including *E. anophelis* and *E. meningoseptica* both before and after the isolation, as well as *E. bruniana* and *E. meningoseptica* both before and after the isolation. These variations warrant further investigation. Studies have shown that most patients with *Elizabethkingia* infections have underlying chronic diseases such as diabetes, cardiovascular diseases, and pulmonary diseases.^20^, ^21^, ^22^^,^^26^, ^27^, ^28^ Our study yielded similar results. Previous studies have shown that *E. meningoseptica* is commonly isolated from ICUs in India^26^ and Taiwan, China.^29^ In the present study, the emergency ICU, trauma ICU, and general ICU were ranked in the top three *Elizabethkingia* sources. These data consistently suggest that *Elizabethkingia* favors infection in immunocompromised patients. In patients infected with *Elizabethkingia*, the mortality rate ranges from 20 % to 40 %.^30^ The major risk factors for patients with *Elizabethkingia* infection include ICU admission, inappropriate antimicrobial therapy, surgery, and the use of an indwelling device.^31^ Other risk factors include COVID-19, prolonged hospitalization, and underlying diseases.^32^ In this study, COVID-19, respiratory illness, advanced age (≥ 65 years), and ICU admission were risk factors for mortality in patients with *Elizabethkingia* infection.

Biofilms are defined as microbial populations composed of bacterial cell aggregates adhered to surfaces and embedded within a self-secreted extracellular matrix. This matrix comprises proteins, extracellular DNA, and polysaccharides.^33^^,^^34^ Bacterial cells within biofilms exhibit high coordination and undergo phenotypic switching, enabling the development of communities resistant to adverse environmental conditions.^35^ This phenotypic switching also contributes to the emergence of antibiotic resistance by facilitating the encoding of resistance genes, inducing genetic mutations, restricting antibiotic penetration, or counteracting host immune defenses.^34^^,^^35^ The persistence of nearly all multidrug-resistant Gram-negative bacteria and their virulence factors during biofilm production represents a significant challenge in hospitalized patients.^36^ Indwelling devices constitute the most critical factor in biofilm formation and colonization.^37^ Biofilms confer protection to bacteria against host immune defenses and antimicrobial agents. Compared to planktonic counterparts, biofilm-forming bacteria demonstrate reduced antibiotic susceptibility and heightened resistance to antimicrobials. *Salmonella enterica*, for instance, forms biofilms on both biotic and abiotic surfaces. This biofilm state enhances bacterial survival through increased antimicrobial resistance and evasion of immune defenses, thereby promoting chronic and device-associated infections.^38^ Studies have confirmed that *Elizabethkingia* form biofilms in moist environments or on water-associated equipment, thereby facilitating their dissemination within hospital settings.^32^^,^^39^ Furthermore, studies have demonstrated a positive association between biofilm formation in *Elizabethkingia* and antibiotic resistance.^16^^,^^40^ Consequently, elucidating the biofilm-forming capacity of *Elizabethkingia* species is imperative. Previous research indicates that *E. meningoseptica* can form biofilms that contaminate hospital environments and cause patient infections, underscoring the need for enhanced environmental disinfection and strict adherence to aseptic procedures by healthcare personnel.^41^ Our study identified *E. anophelis* on environmental surfaces within the ICU, including computer keyboards, mice, chairs, sinks, and hand sanitizer dispenser plungers. Furthermore, a retrospective review of clinical data revealed that *E. anophelis* had also been identified in the sputum of patients admitted to the ward in the previous ten days, leading to the hypothesis that transmission of this bacterium may occur between caregivers and patients and that environmental surfaces and shared medical equipment may also place patients at risk of *Elizabethkingia* infection.^42^ *Elizabethkingia* infections are challenging because they tend to exhibit inherent resistance to antimicrobial agents (including beta-lactams and inhibitors, aminoglycosides, macrolides, tetracycline, vancomycin, and carbapenems) .^3^ Genomic and proteomic analyses have confirmed the presence of multidrug resistance genes and drug efflux systems in *Elizabethkingia*.^43^^,^^44^ Previous studies demonstrated that piperacillin-tazobactam (a β-lactam/β-lactamase inhibitor drug) is relatively sensitive for *E. anopheli*.^1^^,^^45^ However, in the present study, the susceptibility is low (26/90, 28.9 %), compared to *E. meningoceptica* (6/8, 75 %).^1^^,^^46^ This phenomenon is worth exploring in the future. In addition, these strains showed differential susceptibilities to doxycycline, ceftazidime, imipenem, meropenem, amikacin, and tobramycin. The high prevalence of *blaBlaB* and *blaCME* genes in the present study is consistent with broad-spectrum resistance to beta-lactams, including carbapenems. Several genes associated with drug resistance have been identified in *Elizabethkingia*. metallo-beta-lactamase (*MBL*) genes are of global concern as they can confer resistance to carbapenems and almost all β-lactams.^47^ *Elizabethkingia* is the only organism known to carry two distinct *MBL* genes (*blaBlaB* and *blaGOB*) and *blaCME*, which can confer resistance to cephalosporins.^21^ Resistance genes, including *gyrA, gyrB, parC,* and *parE*, and efflux pump genes, including *RND, Major Facilitator Superfamily (MFS), Multidrug and Toxic Compound Extrusion (MATE),* and *ATP-Binding Cassette (ABC)*, were detected in *Elizabethkingia* isolates. The presence of multiple drug resistance genes in *Elizabethkingia* challenges of clinical treatment.^30^

PFGE typing reveals genetic diversity and clonal transmission. Although *E. anophelis* is genotypically highly diverse, clonal transmission has been observed in several pairs of patients from the same or different departments. In contrast to previous studies, our observations over 8 months, from March to November 2023, revealed the presence of 14 patients in the ICU with identical clones of each subtype found in their sputum samples. In three of the ICU admissions (numbered Ean 33, Ean 47, and Ean 76), *E. anophelis* was isolated from the sputum samples within one week. Furthermore, PFGE analysis demonstrated that these samples belonged to the same clone. Based on these findings, we postulate that an outbreak of *E. anophelis* strains occurred in the ICU during the year 2023. From April to August 2022, *E. meningoseptica* isolates were genetically homogeneous (2/7 strains were type A and 2/7 strains were type B) in the hematology and emergency ICU wards, suggesting recent clonal amplification and persistence between the wards. Previous reports have found that the acquisition of *Elizabethkingia* may be associated with water sources or water-related equipment, such as sinks and hand hygiene sink aerators in the hospital environment.^11^^,^^39^ Clonal transmission may be mediated by the hands of hospital staff or patients; Therefore, better hand hygiene and environmental cleanliness are crucial when an isolate is detected in hospitals.^14^ In our study, PFGE J-type clones predominated with 42 isolates identified (50 % of typed specimens), necessitating comprehensive analysis of their transmission dynamics. We propose deploying electronic compliance monitoring systems (e.g., hand hygiene adherence sensors) in J-type clone-endemic wards to correlate hand hygiene event frequency with healthcare worker shift patterns, high-risk procedure intervals (e.g., ventilator adjustments), and temporal-spatial transmission clusters for enhanced traceability. Concurrent weekly ATP bioluminescence testing (RLU < 100 threshold) will be performed on high-touch surfaces—including ventilator interfaces, sinks, and bed rails—with subsequent whole-genome comparative analysis between environmental isolates and clinical J-type clones. Furthermore, healthcare worker movement network modeling will track transmission routes from index cases to incident cases to identify superspreading vectors driving clonal dissemination.

Our study has some limitations. (1) This is a single-center study, which may introduce some bias in the data, and therefore, follow-up studies with larger and more extensive multicenter are needed. (2) We did not further differentiate *Elizabethkingia* isolates by sequencing the *rpoB* gene and by conducting whole genome sequencing. (3) No further investigation into biofilm formation was conducted.

## 5 Conclusions

*Elizabethkingia* infection has become an important public health concern; Therefore, it is crucial to understand its clinical, molecular, and genetic characteristics. In the present study, 16S rRNA gene sequencing was performed on 103 *Elizabethkingia* isolates. Microbiological characterization of the identified *Elizabethkingia* isolates revealed the resistance patterns and genetic diversity of the clinical isolates at this site. As research and clinical practice continue to rely on automated bacteriological identification systems to characterize *Elizabethkingia*, upgrading MALDI-TOF mass spectrometry with expanded reference databases (examples include augmenting biomarker proteins such as ribosomal proteins L29 and L30, S21, and proteins containing the YtxH structural domain^42^), or the use of molecular techniques (such as Whole Genome Sequencing or Next-Generation sequencing^48^), is necessary to accurately characterize these microorganisms. *Elizabethkingia* exhibits variable susceptibility to various antibiotics; Therefore, using antimicrobial susceptibility testing as a guide will enhance the reliability of treatment decisions. Our findings suggest that minocycline has the potential to become the drug of choice for treating *Elizabethkingia* infections, though clinical trials are required. Further research is needed to determine the optimal antimicrobial agents for these life-threatening infections, either alone or in combination.

## Appendix Supplementary materials

Image, application 1

## References

[1] Comba I.Y., Schuetz A.N., Misra A. (2022). Antimicrobial susceptibility of *Elizabethkingia* species: report from a reference laboratory. J Clin Microbiol.

[2] Zajmi A., Teo J., Yeo C.C (2022). Epidemiology and characteristics of *Elizabethkingia spp.* infections in Southeast Asia. Microorganisms.

[3] Lin J.N., Lai C.H., Yang C.H. (2019). *Elizabethkingia* infections in humans: from genomics to clinics. Microorganisms.

[4] King E.O. (1959). Studies on a group of previously unclassified bacteria associated with meningitis in infants. Am J Clin Pathol.

[5] Doijad S., Ghosh H., Glaeser S. (2016). Taxonomic reassessment of the genus *Elizabethkingia* using whole-genome sequencing: *Elizabethkingia endophytica* Kämpfer et al. 2015 is a later subjective synonym of *Elizabethkingia anophelis* Kämpfer. et al. 2011. Int J Syst Evol Microbiol..

[6] Li Y., Kawamura Y., Fujiwara N. (2003). *Chryseobacterium miricola* sp. nov., a novel species isolated from condensation water of space station Mir. Syst Appl Microbiol.

[7] Kämpfer P., Matthews H., Glaeser S.P. (2011). *Elizabethkingia anophelis* sp. nov., isolated from the midgut of the mosquito *Anopheles gambiae*. Int J Syst Evol Microbiol.

[8] Lin I.F., Lai C.H., Lin S.Y. (2023). *In vitro* and *in vivo* antimicrobial activities of vancomycin and rifampin against *Elizabethkingia anophelis*. Int J Mol Sci.

[9] Teo J., Tan S.Y., Tay M. (2013). First case of *E. anophelis* outbreak in an intensive-care unit. Lancet.

[10] Nicholson A.C., Gulvik C.A., Whitney A.M. (2018). Revisiting the taxonomy of the genus Elizabethkingia using whole-genome sequencing, optical mapping, and MALDI-TOF, along with proposal of three novel *Elizabethkingia* species: *Elizabethkingia bruuniana* sp. nov., *Elizabethkingia ursingii* sp. nov., and *Elizabethkingia occulta* sp. nov. *Antonie Van*. Leeuwenhoek.

[11] Lee Y.L., Liu K.M., Chang H.L. (2021). A dominant strain of *Elizabethkingia anophelis* emerged from a hospital water system to cause a three-year outbreak in a respiratory care center. J Hosp Infect.

[12] Johnson W.L., Gupta S.K., Maharjan S. (2024). A genetic locus in *Elizabethkingia anophelis* associated with elevated vancomycin resistance and multiple antibiotic reduced susceptibility. Antibiotics.

[13] Kyritsi M.A., Mouchtouri V.A., Pournaras S. (2018). First reported isolation of an emerging opportunistic pathogen (*Elizabethkingia anophelis*) from hospital water systems in Greece. J Water Health.

[14] Chew K.L., Cheng B., Lin R.T.P. (2018). *Elizabethkingia anophelis* is the dominant *Elizabethkingia* species found in blood cultures in Singapore. J Clin Microbiol.

[15] Wu C., Xiong L., Liao Q.F. (2024). Clinical manifestations, antimicrobial resistance and genomic feature analysis of multidrug-resistant *Elizabethkingia strains*. Ann Clin Microbiol Antimicrob.

[16] Puah S.M., Fong S.P., Kee B.P. (2022). Molecular identification and biofilm-forming ability of *Elizabethkingia* species. Microb Pathog.

[17] Cheng Y.H., Perng C.L., Jian M.J. (2019). Multicentre study evaluating matrix-assisted laser desorption ionization-time of flight mass spectrometry for identification of clinically isolated Elizabethkingia species and analysis of antimicrobial susceptibility. Clin Microbiol Infect.

[18] Han M.S., Kim H., Lee Y. (2017). Relative prevalence and antimicrobial susceptibility of clinical isolates of *Elizabethkingia* species based on 16S rRNA gene sequencing. J Clin Microbiol.

[19] Mahapatra S., Mahapatra A., Sarathi S. (2025). Evaluation of conventional polymerase chain reaction for accurate species identification of *Elizabethkingia* compared to matrix-assisted laser desorption/ionization time-of-flight mass spectrometry. Cureus.

[20] Lin J.N., Lai C.H., Yang C.H. (2018). Comparison of clinical manifestations, antimicrobial susceptibility patterns, and mutations of fluoroquinolone target genes between *Elizabethkingia meningoseptica* and *Elizabethkingia anophelis* isolated in Taiwan. J Clin Med.

[21] Wang L.L., Zhang X.F., Li D. (2020). Molecular characteristics and antimicrobial susceptibility profiles of *Elizabethkingia* clinical isolates in Shanghai, China. Infect Drug Resist.

[22] Chang Y.C., Lo H.H., Hsieh H.Y. (2014). Identification and epidemiological relatedness of clinical *Elizabethkingia meningoseptica* isolates from central Taiwan. J Microbiol Immunol Infect.

[23] Chang Y.C., Lo H.H., Hsieh H.Y. (2015). Identification, epidemiological relatedness, and biofilm formation of clinical *Chryseobacterium indologenes* isolates from central Taiwan. J Microbiol Immunol Infect.

[24] Cheng J., Zhao D.M., Ma X.J. (2023). Molecular epidemiology, risk factors, and outcomes of carbapenem-resistant *Klebsiella pneumoniae* infection in a tertiary hospital in Eastern China: for a retrospective study conducted over 4 years. Front Microbiol.

[25] Dziuban E.J., Franks J.L., So M. (2018). *Elizabethkingia* in children: a comprehensive review of symptomatic cases reported from 1944 to 2017. Clin Infect Dis.

[26] Sengar S., Varghese G., Jamwal A. (2025). *Elizabethkingia anophelis* infection in intensive care unit patients at a tertiary care center in North India: a retrospective study. Am J Trop Med Hyg.

[27] Sarathi S., Behera B., Mahapatra A. (2023). Microbiological characterization and clinical facets of *Elizabethkingia* bloodstream infections in a tertiary care hospital of Eastern India. Infect Drug Resist.

[28] Jiang B., Zhang W.P., Deng N. (2025). A systematic review of reported symptomatic *Elizabethkingia* infection cases in children and adults. Acta Trop.

[29] Huang Y.C., Huang Y.W., Lin Y.T. (2017). Risk factors and outcome of levofloxacin-resistant *Elizabethkingia meningoseptica* bacteraemia in adult patients in Taiwan. Eur J Clin Microbiol Infect Dis.

[30] Burnard D., Gore L., Henderson A. (2020). Comparative genomics and antimicrobial resistance profiling of *Elizabethkingia* isolates reveal nosocomial transmission and *in vitro* susceptibility to fluoroquinolones, tetracyclines, and trimethoprim-sulfamethoxazole. J Clin Microbiol.

[31] Cai Y., Shi Q.X., Yu S.F. (2025). Epidemiological and genomic features of clinical isolates of the *Elizabethkingia* genus in Taizhou City, China. J Glob Antimicrob Resist.

[32] Seong H., Kim J.H., Kim J.H. (2020). Risk factors for mortality in patients with *Elizabethkingia* infection and the clinical impact of the antimicrobial susceptibility patterns of *Elizabethkingia* species. J Clin Med.

[33] Harika K., Shenoy V.P., Narasimhaswamy N. (2020). Detection of biofilm production and its impact on antibiotic resistance profile of bacterial isolates from chronic wound infections. J Glob Infect Dis.

[34] Hashemzadeh M., Dezfuli A.A.Z., Nashibi R. (2021). Study of biofilm formation, structure and antibiotic resistance in *Staphylococcus saprophyticus* strains causing urinary tract infection in women in Ahvaz, Iran. New Microbes New Infect.

[35] Shenkutie A.M., Yao M.Z., Siu G.K. (2020). Biofilm-induced antibiotic resistance in clinical *Acinetobacter baumannii* isolates. Antibiotics.

[36] Husain F.M., Perveen K., Qais F.A. (2021). Naringin inhibits the biofilms of metallo-β-lactamases (MβLs) producing *Pseudomonas* species isolated from camel meat. Saudi J Biol Sci.

[37] Karami N., Lindblom A., Yazdanshenas S. (2020). Recurrence of urinary tract infections with extended-spectrum β-lactamase-producing *Escherichia coli* caused by homologous strains among which clone ST131-O25b is dominant. J Glob Antimicrob Resist.

[38] Muturi P., Mbae C., Kibet E. (2025). Antimicrobial resistance and biofilm formation in rarely reported *Salmonella enterica* serovars from patients presenting with gastroenteritis in Nairobi, Kenya. Front Microbiol.

[39] Choi M.H., Kim M., Jeong S.J. (2019). Risk factors for *Elizabethkingia* acquisition and clinical characteristics of patients, South Korea. Emerg Infect Dis.

[40] Hu S., Lv Y., Xu H. (2022). Biofilm formation and antibiotic sensitivity in *Elizabethkingia anophelis*. Front Cell Infect Microbiol.

[41] Li Y.J., Liu T.T., Shi C.X. (2022). Epidemiological, clinical, and laboratory features of patients infected with *Elizabethkingia meningoseptica* at a tertiary hospital in Hefei City, China. Front Public Health.

[42] Takei S., Teramoto K. (2025). Identification of *Elizabethkingia* species by MALDI-TOF MS proteotyping. Microbiol Spectr.

[43] Spengler G., Kincses A., Gajdács M. (2017). New roads leading to old destinations: efflux pumps as targets to reverse multidrug resistance in bacteria. Molecules.

[44] Agrawal A., Ravikumar R., Varun C.N. (2019). Global proteome profiling reveals drug-resistant traits in *Elizabethkingia meningoseptica*: an opportunistic nosocomial pathogen. OMICS.

[45] Hu R.X., Zhang Q., Gu Z.M. (2020). Molecular diversity of chromosomal metallo-β-lactamase genes in *Elizabethkingia* genus. Int J Antimicrob Agents.

[46] Jian M.J., Cheng Y.H., Chung H.Y. (2019). Fluoroquinolone resistance in carbapenem-resistant *Elizabethkingia anophelis*: phenotypic and genotypic characteristics of clinical isolates with topoisomerase mutations and comparative genomic analysis. J Antimicrob Chemother.

[47] Chang T.Y., Chen H.Y., Chou Y.C. (2019). *In vitro* activities of imipenem, vancomycin, and rifampicin against clinical *Elizabethkingia* species producing BlaB and GOB metallo-beta-lactamases. Eur J Clin Microbiol Infect Dis.

[48] Wang W., Chauhan V., Luo Y.T. (2024). Comparing NGS-based identification of bloodstream infections to traditional culture methods for enhanced ICU care: a comprehensive study. Front Cell Infect Microbiol.

