# Supplementary material for: Prevalence, microbiological features, and clinical characteristics of Elizabethkingia isolates in a tertiary hospital, Jiangxi Province, China
Source: Infect Med (Beijing). 2025 Aug 19;4(3):100198. doi: 10.1016/j.imj.2025.100198 (PMC12451349; doi:10.1016/j.imj.2025.100198)
Supplement: Supplementary file 1 [file mmc1.docx]

Supplementary material

Table S1 Clinical characteristics of the 103 *Elizabethkingia* isolates.

| Characteristics, *n* (%) | All (*n* = 103) | Number of episodes (%) | | | |
| --- | --- | --- | --- | --- | --- |
|  |  | *E. anophelis* (*n* = 92) | *E. meningoseptic* (*n* = 8) | *E. bruuniana* (*n* = 2) | *E. ursingii* (*n* = 1) |
| Admission to intensive care unit | 88 (85.4) | 81 (88.0) | 5 (62.5) | 2 (100.0) | 0 |
| Shock | 28 (27.2) | 26 (28.3) | 1 (12.5) | 1 (50.0) | 0 |
| COVID-19 | 11 (10.7) | 10 (10.9) | 0 | 1(50.0) | 0 |
| Case fatality | 36 (35.0) | 33 (35.9) | 2 (25.0) | 1 (50.0) | 0 |
| Nasogastric tube | 73 (70.9) | 66 (71.7) | 6 (75.0) | 1 (50.0) | 0 |
| Foley's catheter | 80 (77.7) | 72 (78.3) | 6 (75.0) | 1 (50.0) | 1 (100.0) |
| Surgical puncture or drain | 47 (45.6) | 43 (46.7) | 3 (37.5) | 1 (50.0) | 0 |
| Underlying diseases | | | | | |
| Diabetes mellitus | 18 (17.5) | 15 (16.3) | 2 (25.0) | 1 (50.0) | 0 |
| Hypertension | 37 (35.9) | 31 (33.7) | 4 (50.0) | 2 (100.0) | 0 |
| Chronic obstructive pulmonary disease | 11 (10.7) | 11 (12.0) | 0 | 0 | 0 |
| Principle disease | | | | | |
| Nervous system | 61 (59.2) | 53 (57.6) | 6 (75.0) | 2 (100.0) | 0 |
| Malignancy | 5 (4.9) | 4 (4.3) | 1 (12.5) | 0 | 0 |
| Trauma | 40 (38.3) | 37 (40.2) | 3 (37.5) | 0 | 0 |
| Cardiovascular | 43 (41.7) | 36 (39.1) | 5 (62.5) | 2 (100.0) | 0 |
| Digestive | 21 (20.4) | 19 (20.7) | 2 (25.0) | 0 | 0 |
| Respiratory | 35 (34.0) | 32 (34.8) | 1 (12.5) | 2 (100.0) | 0 |

Table S2 Risk factors associated with in-hospital mortality

| Factor, *n* (%) | Multivariate analysis | |
| --- | --- | --- |
|  | 95% CI | *p* |
| Age ≥ 65 years | 1.909 (0.497–7.337) | 0.347 |
| Admission to intensive care unit | − | 0.999 |
| COVID-19 | 8.000(1.001–63.963) | 0.050 |
| Chronic obstructive pulmonary | 0.917 (0.166–5.048) | 0.920 |
| Mechanical ventilation | 0.214 (0.037–1.234) | 0.085 |

Fig. S1. Recurrent isolation counts of *Elizabethkingia* *spp*. per patient.
